# Run-time Complexity Bounds Using Squeezers

**DOI:** 10.1007/978-3-030-72019-3_12

**Published:** 2021-03-23

**Authors:** Oren Ish-Shalom, Shachar Itzhaky, Noam Rinetzky, Sharon Shoham

**Affiliations:** Imperial College, London, UK; 9 Tel Aviv University, Tel Aviv, Israel; 10 Technion, Haifa, Israel

## Abstract

Determining upper bounds on the time complexity of a program is a fundamental problem with a variety of applications, such as performance debugging, resource certification, and compile-time optimizations. Automated techniques for cost analysis excel at bounding the resource complexity of programs that use integer values and linear arithmetic. Unfortunately, they fall short when execution traces become more involved, esp. when data dependencies may affect the termination conditions of loops. In such cases, state-of-the-art analyzers have shown to produce loose bounds, or even no bound at all.

We propose a novel technique that generalizes the common notion of recurrence relations based on ranking functions. Existing methods usually unfold one loop iteration, and examine the resulting relations between variables. These relations assist in establishing a recurrence that bounds the number of loop iterations. We propose a different approach, where we derive recurrences by comparing *whole traces* with *whole traces* of a lower rank, avoiding the need to analyze the complexity of intermediate states. We offer a set of global properties, defined with respect to whole traces, that facilitate such a comparison, and show that these properties can be checked efficiently using a handful of local conditions. To this end, we adapt *state squeezers*, an induction mechanism previously used for verifying safety properties. We demonstrate that this technique encompasses the reasoning power of bounded unfolding, and more. We present some seemingly innocuous, yet intricate, examples where previous tools based on *cost relations* and control flow analysis fail to solve, and that our squeezer-powered approach succeeds.

### 1 Introduction

Cost analysis is the problem of estimating the resource usage of a given program, over all of its possible executions. It complements functional verification—of safety and liveness properties—and is an important task in formal software certification. When used in combination with functional verification, cost analysis ensures that a program is not only correct, but completes its processing in a reasonable amount of time, uses a reasonable amount of memory, communication bandwidth, etc. In this work we focus on run-time complexity analysis. While the area has been studied extensively, e.g., [19], [28], [3], [14], [6], [16], [21], [12], [9], the general problem of constraining the number of iterations in programs containing loops with arbitrary termination conditions remains hard.

A prominent approach to computing upper bounds on the time complexity of a program identifies a well-founded numerical measure over program states that decreases in

```
void binary_counter(unsigned int n) {
  unsigned int c[n];
  memset(c,0,n*sizeof(unsigned int));
  int i=0;
  while (i < n) {
    if (c[i] == 1) /*scan 1-prefix*/{c[i] = 0; i++;          }
    else           /*increment*/    {c[i] = 1; i=0; print(c);}
  }}
```

**Fig. 1.** A program that produces all combinations of $n$ bits.

every step of the program, also called a *ranking function*. In this case, an upper bound on the measure of the initial states comprises an upper bound on the program's time complexity. Finding such measures manually is often extremely difficult. The *cost relations* approach, dating back to [28], attempts to automate this process by using the control flow graph of the program to extract recurrence formulas that characterize this measure. Roughly speaking, the recurrences relate the measures (costs) of adjacent nodes in the graph, taking into account the cost of the step between them. In this way, the cost relations track the evolution of the measure between *every* pair of consecutive states along the executions of the program.

One limitation of cost relations is the need to capture the number of steps remaining for execution in *every* state, that is, all intermediate states along all executions. If the structure of the state is complex, this may require higher order expressions, e.g., summing over an unbounded number of elements. As an example, consider the program in Fig. 1 that implements a binary counter represented by an array of bits.

In this case, a ranking function that decreases between every two consecutive iterations of the loop, or even between two iterations that print the value of the counter, depends on the *entire* content of the array. Attempting to express a ranking function over the scalar variables of this program is analogous to abstracting the loop as a finite-state system that ignores the content of the array, and as such contains transition cycles (e.g. the abstract state $\langle n \mapsto n_0, i \mapsto 0\rangle$, obtained by projecting the state to the scalar variables only, repeats multiple times in any trace)—meaning that no strictly decreasing function can be defined in this way. Similarly, any attempt to consider a bounded number of bits will encounter the same difficulty.

In this paper, we propose a novel approach for extracting recurrence relations capturing the time complexity of an imperative program, modeled as a transition system, by relating whole traces instead of individual states. The key idea is to relate a trace to (one or more) shorter traces. This allows to formulate a recurrence that resolves to the length of the trace and recurs over the values at the initial states only. We sidestep the need to take into account the more complex parts of the state that change along the trace (e.g., in the case of the binary counter, the array is initialized with zeros).

Our approach relies on the notion of *state squeezers* [22], previously used exclusively for the verification of safety properties. We present a novel aspect where the same squeezers can be used to determine complexity bounds, by replacing the safety property check with trace length judgements.

Squeezers provide a means to perform induction on the "size" of (initial) states to prove that all reachable states adhere to a given specification. This is accomplished by attaching *ranks* from a well-founded set to states, and defining a *squeezer function* that maps states to states of a lower rank. Note that the notion of a rank used in our work is distinct from that of a ranking function, and the two should not be confused; in particular, a rank is not required to decrease on execution steps. Previously, squeezers were utilized for safety verification: the ability to establish safety is achieved by having the squeezer map states in a way that forms a (relaxed form of) a *simulation relation*, ensuring that the traces of the lower-rank states simulate the traces of the higher rank states. Due to the simulation property, which is verified locally, safety over states with a *base* rank, carries over (by induction over the rank) to states of any higher rank.

In this work, we use the construction of well-founded ranks and squeezers to define a *recurrence formula* representing (an upper bound on) the time complexity of the procedure being analyzed. We do so by expressing the complexity (length) of traces in terms of the complexity of lower-rank traces. This new setting raises additional challenges: it is no longer sufficient to relate traces to lower-rank traces; we also need to *quantify the discrepancy* between the lengths of the traces, as well as between their ranks. This is achieved by a certain form of simulation that is parameterized by *stuttering shapes* (for the lengths) and by means of a *rank bounding function* (for the ranks). Furthermore, while [22] limits each trace to relate to a *single* lower-rank trace, we have found that it is sometimes beneficial to employ a *decomposition* of the original trace into *several* consecutive *trace segments*, so that each segment corresponds to *some* (possibly different) lower-rank trace.The segmentation simplifies the analysis of the length of the entire trace, since it creates sub-analyses that are easier to carry out, and the sum of which gives the desired recurrence formula. This also enables a richer set of recurrences to be constructed automatically, namely non-single recurrences (meaning that the recursive reference may appear more than once on the right hand side of the equation).

The base case of the recurrence is obtained by computing an upper bound on the time complexity of base-rank states. This is typically a simpler problem that may be addressed, e.g., by symbolic execution due to the bounded nature of the base. The solution to the recurrence formula with the respective base case soundly overapproximates the time complexity of the procedure.

We show that, conceptually, the classical approach for generating recurrences based on ranking functions can be viewed as a special case of our approach where the squeezer maps a state to its immediate successor. The real power of our approach is in the freedom to define other squeezers, producing simpler recursions, and avoiding the need for complex ranking functions.

Our use of squeezers for extracting recurrences that bound the complexity of imperative programs is related to the way analyses for functional programs (e.g. [20]) use the term(s) in recursive function calls to extract recurrences. The functional programming style coincidentally provides such candidate terms. The novelty of our approach is in introducing the concept of a squeezer explicitly, leading to a more flexible analysis as it does not restrict the squeezer to follow specific terms in the program. In particular, this allows reasoning over space in imperative programs as well.

The main results of this paper can be summarized as follows:

- We propose a novel technique for run-time complexity analysis of imperative programs based on state squeezers. Squeezers, together with rank-bounding functions, are used for extracting recurrence relations whose solutions overapproximate the length of executions of the input program.
- We formalize the notions of *state squeezers*, *partitioned simulation* and *rank bounding functions* that underlie the approach, and establish conditions that ensure soundness of the recurrence relations.
- We demonstrate that squeezers and rank bounding functions can be efficiently synthesized and verified, due to their compactness, especially relative to explicit ranking functions.
- We implemented our approach and applied it successfully to several small but intricate programs, some of which could not have been handled by existing techniques.

### 2 Overview

In this section we give a high level description of our technique for complexity analysis using the binary counter example in Fig. 1.

*Example: Binary counter* The procedure in Fig. 1 receives as an input a number $n$ of bits and iterates over all their possible values in the range $0...2^n - 1$. The "current" value is maintained in an array $c$ which is initialized to zero and whose length is $n$. $c[0]$ represents the least significant bit. The loop scans the array from the least significant bit forward looking for the leftmost 0 and zeroing the prefix of 1s. As soon as it encounters a 0, it sets it to 1 and starts the scan from the beginning. The program terminates when it reaches the end of the array $(i = n)$, all array entries are zeros, and the last value was $111\ldots$; at this point all the values have been enumerated.

*Existing analyses* All recent methods that we are aware of (such as [16,4,20]) fail to analyze the complexity of this procedure (in fact, most methods will fail to realize that the loop terminates at all). One reason for that is the need to model the contents of the array whose size in unknown at compile time. However, even if data *were* modeled somehow and taken into account, finding a ranking function, which underlies existing approaches, is hard since this function is required to decrease between *any* two consecutive iterations along *any* execution. Here for instance, to the best of our knowledge, such a function would depend on an unbounded number of elements of the array; it would need to extract the current value as an integer, along the lines of $\sum_{j=0}^{n-1} c[j] \cdot 2^j$.

The use of a ranking function for complexity analysis is somewhat analogous to the use of inductive invariants in safety verification. Both are based on induction over time along an execution. This paper is inspired by previous work [22] showing that verification can also be done when the induction is performed on the size (*rank*) of the state rather than on the number of iterations, where the size of the state may correspond, e.g., to the size of an unbounded data structure. We argue that similar concepts can be applied in a framework for complexity classification. That is, we try to infer a recurrence relation that is *based on the rank* of the state and correlates the lengths of *complete* executions—executions that start from an initial state—of different ranks. This sidesteps the need to express the length of *partial* executions, which start from intermediate states. While the approach applies to bounded-state systems as well, its benefits become most apparent when the program contains a-priori unbounded stores, such as arrays.

*Our approach.* Roughly speaking, our approach for computing recurrence formulas that provide an upper bound on the complexity of a procedure is based on the following ingredients:

- A *rank* function $r : init \to X$ that maps initial states to ranks from a well founded set $(X, \prec)$ with base $B$. Intuitively, the rank of the initial state governs the time complexity of the entire trace, and we also consider it to be the rank of the trace. As we shall soon see, this rank can be significantly simpler than a ranking function.
- A *squeezer* $\curlyvee : \Sigma \to \Sigma$ that maintains (some variant of) a simulation relation, thus ensuring a bona fide correspondence between higher-rank traces and lower-rank traces through correspondence between states.
- A *trace partition* $p_d : \Sigma \to [1..d]$ that maps each state to a segment-identifier $i \in [1..d]$, and induces a decomposition of a trace into *segments*, allowing $\curlyvee$ to map each of them to a separate, lower-rank *mini-trace*.
- A *rank-bounding* function $\hat{\curlyvee} : X \times [1..d] \to X$ that provides an upper bound on the rank of the initial states of the $d$ mini-traces based on the rank of the higher-rank trace. (The rank is *not* required to be uniform across mini-traces).

All of these ingredients are synthesized automatically, as we discuss in Section 4. Next, we elaborate on each of these ingredients, and illustrate them using the binary counter example. We further demonstrate how we use these ingredients to find recurrence formulas describing (an upper bound on) the complexity of the program.

*Some notations* We adopt a standard encoding of a program as a transition system over a state space $\Sigma$, with a set of initial states $init \subseteq \Sigma$ and transition function $tr : \Sigma \to \Sigma$, where a transition corresponds to a loop iteration. We use $reach \subseteq \Sigma$ to denote the set of reachable states, $reach = \{\sigma \mid \exists \sigma_0, k.\ tr^k(\sigma_0) = \sigma \wedge \sigma_0 \in init\}$.

*Defining the rank of a state* Ranks are taken from a well founded set $(X, \prec)$ with a basis $B \subseteq X$ that contains all the minimal elements of $X$. The rank function, $r : init \to X$, aims to abstract away irrelevant data from the (initial) state that does *not* effect the execution time, and only uses state "features" that do. When proper ranks are used, the rank of an initial state is all that is needed to provide a tight bound on its trace length. Since ranks are taken from a well founded set, they can be recursed over. In the binary counter example, the chosen rank is $n$, namely, the rank function maps each state to the size of the array. (Notice that the rank does not depend on the contents of the array; in contrast, bounding the trace length from any intermediate state, and not just initial states, would have required considering the content of the array).

Given the rank function, our analysis extracts a recurrence formula for the complexity function $comp_x : X \to \mathbb{N} \cup \{\infty\}$ that provides an upper bound on the number of iterations of $tr$ based on the rank of the *initial states*. In our exposition, we sometimes

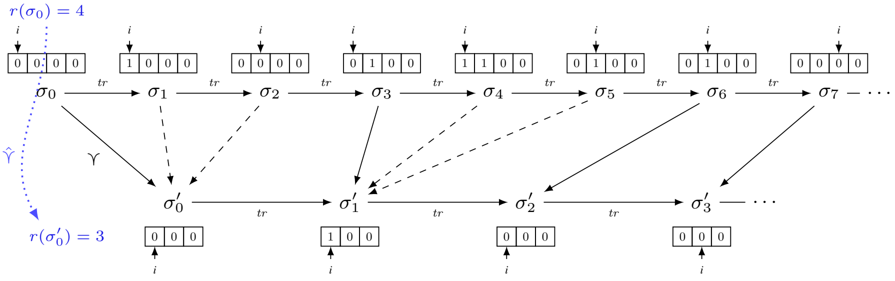

$\Upsilon\big(\langle n, i, c\rangle\big) = \langle n \dot{-} 1, i \dot{-} 1, c[1{:}]\rangle$ $\hat{\Upsilon}(n) = n \dot{-} 1$ $\big(x \dot{-} y = \max\{0, x - y\}\big)$

**Fig. 2.** Correspondence between two traces of the binary counter program. Squeezer removes the leftmost array entry, that represents the least significant bit. The rank is the array size, i.e., four on the upper trace and three on the lower one. The simulation includes only 1-,2- and 3-steps, so the length of the upper trace is at most three times that of the lower trace, yielding an overall complexity bound of $O(3^n)$.

also refer to a time complexity function over states, $comp_s : init \to \mathbb{N} \cup \{\infty\}$, which is defined directly on the (initial) states, as the number of iterations in an execution that starts with some $\sigma_0 \in init$.

*Defining a squeezer* The squeezer $\Upsilon : \Sigma \to \Sigma$ is a function that maps states to states of lower-rank traces (where the rank of a trace is determined by the rank of its initial state), down to the base ranks $B$. Its importance is in defining a correspondence between higher-rank traces and lower-rank ones that can be verified locally, by examining individual states rather than full traces. The kind of correspondence that the squeezer is required to ensure affects the flexibility of the approach and the kind of recurrence formulas that it may yield. To start off, consider a rather naive squeezer that satisfies the following local properties:

– rank decrease of non-base initial states: $\sigma_0 \in init \wedge r(\sigma_0) \notin B \Rightarrow r(\Upsilon(\sigma_0)) \prec r(\sigma_0)$, and
– simulation
  • initial anchor: $\sigma_0 \in init \Rightarrow \Upsilon(\sigma_0) \in init$,
  • $k$-step: $\sigma \in reach \Rightarrow \exists k.\ tr(\Upsilon(\sigma)) = \Upsilon(tr^k(\sigma))$.

As an example, the squeezer we consider for the binary counter program is rather intuitive: it removes the least significant bit ($c[0]$), and adjusts the index $i$ accordingly. Doing so yields a state with rank $r(\Upsilon(\sigma_0)) = r(\sigma_0) - 1$. Fig. 2 shows the correspondence between a 4-bit binary counter, and a 3-bit one. The figure illustrates the simulation $k$-step property for $k = 1, 2, 3$: $\sigma_0$ and $\sigma_3$ are $(3, 1)$-stuttering, $\sigma_1$ and $\sigma_4$ are $(2, 1)$-stuttering, and $\sigma_2$, $\sigma_5$ and $\sigma_6$ are $(1, 1)$-stuttering.

The simulation property induces a correlation between a higher rank trace $\tau$ and a lower rank one $\tau'$, such that every step of $\tau'$ is matched by $k$ steps in $\tau$. Whenever a state $\sigma$ satisfies the $k$-step property, we will refer to it as being $(k, 1)$-*stuttering*. (We usually only care about the smallest $k$ that satisfies the property for a given $\sigma$.) Now suppose that there exists some $\widehat{k} \in \mathbb{N}^+$ such that for every trace $\tau(\sigma_0)$ and every state

$\sigma \in \tau(\sigma_0)$, $\sigma$ is $(k, 1)$-stuttering with $1 \leq k \leq \widehat{k}$. This would yield the following complexity bound:

$$comp_s(\sigma_0) \leq \widehat{k} \cdot comp_s(\Upsilon(\sigma_0)). \tag{1}$$

*All your base* [3] What should happen if we repeatedly apply $\Upsilon$ to some initial state $\sigma_0$, each time obtaining a new, lower-rank trace? Since $r(\Upsilon(\sigma_0)) \prec r(\sigma_0)$, and since $(X, \prec)$ is well-founded, we will eventually hit some state of *base rank*:

$$\Upsilon(\Upsilon(\ldots(\sigma_0))\ldots) = \sigma_0^\circ \quad \text{such that} \quad r(\sigma_0^\circ) \in B$$

Hence, if we know the complexity of the initial states with a base rank, we can apply Eq. (1) iteratively to compute an upper bound of the complexity of *any* initial state.

How many steps will be needed to get from an arbitrary initial state $\sigma_0$ to $\sigma_0^\circ$? Clearly, this depends on the rank, and the way in which $\Upsilon$ decreases it.

Consider the binary counter program again, with the rank $r(\sigma) = n$. $(\mathbb{N}, <)$ is well-founded, with a single minimum 0. If we define, e.g., $B = \{0, 1\}$, we know that the length of any trace with $n \in B$ is bounded by a constant, 2. (Bounding the length of traces starting from an initial state $\sigma_0$ where $r(\sigma_0) \in B$ can be done with known methods, e.g., symbolic execution). Since the rank decreases by 1 on each "squeeze", we get the following exponential bound:

$$comp_s(\sigma_0) \leq 2 \cdot 3^{n-1} = O(3^n) \tag{2}$$

The last logical step, going from (1) to (2), is, in fact, highly involved: since Eq. (1) is a mapping of *states*, solving such a recurrence for arbitrary $\Upsilon$ cannot be carried out using known automated methods. Instead, we implicitly used the rank of the state, $n$, to extract a recurrence over scalar values and obtain a closed-form expression. Let us make this reasoning explicit by first expressing Eq. (1) in terms of $comp_x$ instead of $comp_s$:

$$comp_x(n) \leq \widehat{k} \cdot comp_x(n-1)$$

Here, $n - 1$ denotes the rank obtained when squeezing an initial state of rank $n$. Unlike Eq. (1), this is a recurrence formula over $(\mathbb{N}, <)$ that may be solved algorithmically, leading to the solution $comp_x(n) = O(3^n)$.

*Surplus analysis* Assuming the worst $k$ for all the states in the trace can be too conservative; in particular, if there are only a few states that satisfy the $\widehat{k}$-step property, and all the others satisfy the 1-step property. In the latter case, if we know that at most $b$ states in any one trace have $k > 1$, we can formulate the tighter bound:

$$comp_s(\sigma_0) \leq comp_s(\Upsilon(\sigma_0)) \; + \; \widehat{k} \cdot b \tag{3}$$

Incidentally, in the current setting of the binary counter program, the number of $\widehat{k}$-steps (3-steps) is *not* bounded. So we cannot apply the inequality (3) repeatedly on any trace, as the number of 3-steps depends on the initial state. However, we can improve the analysis by partitioning the trace to two parts, as we explain next.

[3] `https://knowyourmeme.com/memes/all-your-base-are-belong-to-us`

*Segments and mini-traces* Note that both (1) and (3) "suffer" from an inherent restriction that the right hand side contains *exactly* one recursive reference. As such, they are limited in expressing certain kinds of complexity classes.

In order to get more diverse recurrences, including non-single recurrences, we propose an extension of the simulation property that allows more than one lower-rank trace:

- *partitioned* simulation
  - initial anchor: $\sigma_0 \in init \Rightarrow \curlyvee(\sigma_0) \in init$ *(same as before)*,
  - $k$-step: $\sigma \in reach \Rightarrow \exists k.\ tr\big(\curlyvee(\sigma)\big) = \curlyvee\big(tr^k(\sigma)\big)$ *(same as before)* <u>*or*</u>
    $\curlyvee\big(tr(\sigma)\big) \in init$ *(switch)*

This definition allows a new mini-trace to start at any point along a higher-rank trace $\tau$, thus marking the beginning of a new segment of $\tau$. When this occurs, we call $tr(\sigma)$ a *switch state*. For the sake of uniformity, we also refer to all initial states $\sigma_0 \in init$ as switch states. Hence, each segment of $\tau$ starts with a switch state, and the mini-traces are the lower-level traces that correspond to the segments (these are the traces that start from $\curlyvee(\sigma_s)$, where $\sigma_s$ is a switch state). The length of $\tau$ can now be expressed as the *sum* of lower-level mini-traces.

However, there are two problems remaining. First, we need to extend the "rank decrease of non-base initial states" requirement to any switch state in order to ensure that the ranks of all mini-traces are indeed lower. Namely, we need to require that if $\sigma_s$ is any switch state in a trace from $\sigma_0$, then $r\big(\curlyvee(\sigma_s)\big) \prec r(\sigma_0)$. Second, even if we extend the rank decrease requirement, this definition does not suggest a way to bound the number of correlated mini-traces and their respective ranks, and therefore suggests no effective way to produce an equation for $comp_s$ as before.

To sidestep the problem of a potentially unbounded number of mini-traces, we augment the definition of simulation with a *trace partition* function; to address the challenge of the rank decrease we use a *rank-bounding* function, which is responsible both for ensuring that the rank of the mini-traces decreases and for bounding their ranks.

*Defining a partition* We define a function $p_d : \Sigma \to \{1, \ldots, d\}$, parameterized by a constant $d$, called a *partition function*, that is weakly monotone along any trace ($p_d(\sigma) \leq p_d(tr(\sigma))$). This function induces a partition of any trace $\tau$ into (at most) $d$ segments by grouping states based on the value of $p_d(\sigma)$. To ensure the segments and mini-traces are aligned, we require that switch states only occur at segment boundaries.

- *d-partitioned* simulation:
  - initial anchor: $\sigma_0 \in init \Rightarrow \curlyvee(\sigma_0) \in init$ *(same as before)*,
  - $k$-step: $\sigma \in reach \Rightarrow \exists k.\ tr\big(\curlyvee(\sigma)\big) = \curlyvee\big(tr^k(\sigma)\big)$ *(same as before)* <u>*or*</u>
    $\curlyvee\big(tr(\sigma)\big) \in init \land p_d(\sigma) < p_d\big(tr(\sigma)\big)$ *(segment switch)*

In our running example, let us change $\curlyvee$ so that it shrinks the state by removing the *most* significant bit instead of the least. This leads to a partition of the execution trace for $r(\sigma_0) = n$ into two segments, as shown in Fig. 3. The partition function is $p_d = (i \geq n \mid\mid c[n-1])\ ?\ 2\ :\ 1$ (essentially, $c[n-1]+1$, except that the final state is slightly different). As can be seen from the figure, each segment simulates a mini-trace

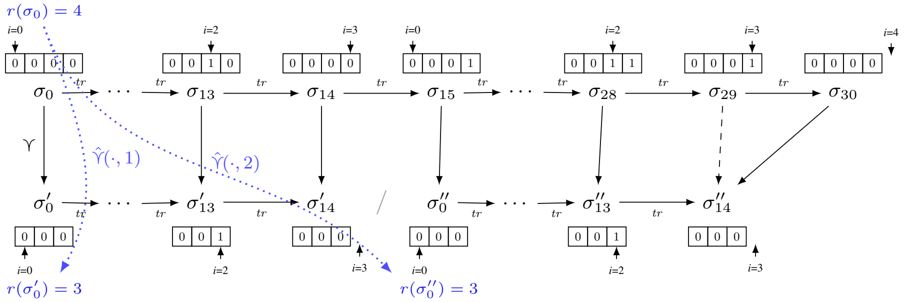

$\Upsilon\big(\langle n, i, c\rangle\big) = \langle n \dot{-} 1, (i < n) \; ? \; i : i{-}1, c[:n{-}1]\rangle \qquad \hat{\Upsilon}(n, 1) = n \dot{-} 1$

**Fig. 3.** An execution trace of the binary counter program that corresponds to two mini-traces of lower rank.

of rank $n - 1$, with $k = 1$ for all the steps except for the last step (at $\sigma_{28}$) where $k = 2$. In this case, it would be folly to use the recurrence (1) with $\widehat{k} = 2$, since all the steps are 1:1 except one. Instead, we can formulate a tighter bound:

$$comp_s(\sigma_0) \leq comp_s(\sigma_0') + comp_s(\sigma_0'') + 2$$

Where: $comp_s(\sigma_0')$, $comp_s(\sigma_0'')$ are the lengths of the mini-traces, and 2 is the surplus from the switch transition $\sigma_{14} \to \sigma_{15}$ plus the 2-step at $\sigma_{28}$. In the case of this program, we know that $r(\sigma_0') = r(\sigma_0'') = r(\sigma_0) - 1$, for any initial state $\sigma_0$, therefore, turning to $comp_x$, we can derive and solve the recurrence $comp_x(n) = 2 \cdot comp_x(n{-}1) {+} 2$, which together with the base yields the bound:

$$comp_x(n) = 2^{n+1} - 2$$

Clearly, a general condition is required in order to identify the ranks of the corresponding initial states of the (lower-rank) mini-traces (and at the same time, ensure that they decrease).

*Bounding the ranks of squeezed switch states* This is not a trivial task, since as previously noted, the squeezed ranks could be different, and may depend on properties present in the corresponding switch states. To achieve this goal, once a partition function $p_d$ is defined, we also define a rank-bounding function $\hat{\Upsilon} : X \times \{1, \ldots, d\} \to X$, where for any $\sigma_0 \in init$ and switch state $\sigma_s$, $\hat{\Upsilon}$ provides a bound for the rank of $\Upsilon(\sigma_s)$ based on that of $\sigma_0$:

$$r(\Upsilon(\sigma_s)) \preceq \hat{\Upsilon}\big(r(\sigma_0), p_d(\sigma_s)\big) \prec r(\sigma_0) \tag{4}$$

The rightmost inequality ensures that a mini-trace that starts from $\Upsilon(\sigma_s)$ is of lower-rank than $\sigma_0$, and as such extends the "rank decrease" requirement to all mini-traces. Based on this restriction, we can formulate a recurrence for $comp_x$ based on the initial rank $\rho = r(\sigma_0)$, as follows:

$$comp_x(\rho) \leq \sum_{i=1}^{d} comp_x\big(\hat{\Upsilon}(\rho, i)\big) \; + \; (d-1) \; + \; \widehat{k} \cdot b \tag{5}$$

Where $b$, as before, is the number of $k$-steps for which $k > 1$, and $\widehat{k}$ is the bound on $k$ ($k \leq \widehat{k}$). The expression $(d-1)$ represents the transitions between segments, and $\widehat{k} \cdot b$ represents the surplus of the $\rho$-rank trace over the total lengths of the mini-traces.

It should be clear from the definition above, that $\hat{\curlyvee}$ is quite intricate. How would we compute it effectively? The rank decrease of the initial states and the simulation properties were *local* by nature, and thus amenable to validation with an SMT solver. The $\hat{\curlyvee}$ function is inherently *global*, defined w.r.t. an entire trace. This makes the property (4) challenging for verification methods based on SMT. To render this check more feasible with first-order reasoning, we introduce two special cases where the problem of checking (4) becomes easier: rank preservation and a single segment, explained next.

*Taming $\hat{\curlyvee}$ with rank preservation* To obtain rank preservation, we extend the rank function to all states (instead of just the initial states), and require that the rank is preserved along transitions. This is appropriate in some of the scenarios we encountered. For example, the binary counter illustration satisfies the property that along any execution $\{\sigma_i\}_{i=0}^{\infty}$, the rank is preserved: $r(\sigma_i) = r(\sigma_{i+1})$. Rank preservation means that given a switch state $\sigma_s$ of an arbitrary segment $i$, we know that $r(\sigma_s) = r(\sigma_0)$. Once this is set, $\hat{\curlyvee}$ only needs to overapproximate the rank of $\curlyvee(\sigma_s)$ in terms of the rank of the same state $\sigma$.

*Taming $\hat{\curlyvee}$ with a single segment* In this case, checking (4) reduces to a single check of the initial state, which is the only switch state. It turns out that the restriction to a single segment is still expressive enough to handle many loop types.

*Putting it all together* Theoretically, $r$, $\curlyvee$, $p_d$, and $\hat{\curlyvee}$ can be manually written by the user. However, this is a rather tedious task, that is straightforward enough to be automated. We observed that all the aforementioned functions are simple enough entities, that can be expressed through a strict syntax using first order logic. Similar to [22], we apply a generate-and-test synthesis procedure to enumerate a space of possible expressions representing them. This process is explained in Section 4.

### 3 Complexity Analysis based on Squeezers

In this section we develop the formal foundations of our approach for extracting recurrence relations describing the time complexity of an imperative program based on state squeezers. We present the ingredients that underly the approach, the conditions they are required to satisfy, and the recurrence relations they induce. In the next section, we explain how to extract the recurrences automatically. Given the recurrence relation, a dedicated (external) tool may be applied to end up with a closed formula, similar to [3].

We use *transition systems* to capture the semantics of a program.

**Definition 1 (Transition Systems).** *A transition system is a tuple* $(\Sigma, init, tr)$*, where* $\Sigma$ *is a set of* states*,* $init \subseteq \Sigma$ *is a set of* initial states *and* $tr : \Sigma \to \Sigma$ *is a* transition function *(rather than a transition relation, since only deterministic procedures are*

*considered). The set of* terminal states $F \subseteq \Sigma$ *is implicitly defined by* $tr(\sigma) = \sigma$. *An* execution trace *(or a* trace *in short) is a finite or infinite sequence of states* $\tau = \sigma_0, \sigma_1, \ldots$ *such that* $\sigma_{i+1} = tr(\sigma_i)$ *for every* $0 \leq i < |\tau|$. *A state* $\sigma \in \Sigma$ *defines an execution trace* $\tau(\sigma) = \{tr^i(\sigma)\}_{i \in \mathbb{N}}$. *Whenever there exists an index* $0 \leq k \leq |\tau|$ *s.t.* $\sigma_k \in F$, *we truncate* $\tau(\sigma)$ *into a finite trace* $\{tr^i(\sigma)\}_{i=0}^k$, *where* $k$ *is the minimal such index. The trace is* initial *if it starts from an initial state, i.e.,* $\sigma \in init$. *Unless explicitly stated otherwise, all traces we consider are initial. The set of* reachable states *is* $reach = \{\sigma \in \Sigma \mid \exists \sigma_0 \in init \, . \, \sigma \in \tau(\sigma_0)\}$.

Roughly, to represent a program by a transition system, we translate it into a single loop program, where $init$ consists of the states encountered when entering the loop, and transitions correspond to iterations of the loop.

In the sequel, we fix a transition system $(\Sigma, init, tr)$ with a set $F$ of terminal states and a set $reach$ of reachable states.

**Definition 2 (Complexity over states).** *For a state* $\sigma \in \Sigma$, *we denote by* $comp_s(\sigma)$ *the number of transitions from* $\sigma$ *to a terminal state along* $\tau(\sigma)$ *(the trace that starts from* $\sigma$*). Formally, if* $\tau(\sigma)$ *does not include a terminal state, i.e., the procedure does* not *terminate from* $\sigma$, *then* $comp_s(\sigma) = \infty$. *Otherwise:*

$$comp_s(\sigma) = \min\{k \in \mathbb{N} \mid tr^k(\sigma) \in F\}.$$

*The complexity function of the program maps each initial state* $\sigma_0 \in init$ *to its time complexity* $comp_s(\sigma_0) \in \mathbb{N} \cup \{\infty\}$.

Our complexity analysis derives a recurrence relation for the complexity function by expressing the length of a trace in terms of the lengths of traces that start from lower rank states. This is achieved by (i) attaching to each initial state a *rank* from a well-founded set that we use as the argument of the complexity function and that we recur over, and (ii) defining a *squeezer* that maps each state from the original trace to a state in a lower-rank trace; the mapping forms a *partitioned simulation* according to a *partition function* that decomposes a trace to segments; each segment is simulated by a (separate) lower-rank trace, allowing to express the length of the former in terms of the latter, and finally, (iii) defining a *rank bounding function* that expresses (an upper bound on) the ranks of the lower-rank traces in terms of the rank of the higher-rank trace. We elaborate on these components next.

#### 3.1 Time complexity as a function of rank

We start by defining a rank function that allows us to express the time complexity of an initial state by means of its rank.

**Definition 3 (Rank).** *Let* $X$ *be a set, and* $\prec$ *be a well-founded partial order over* $X$. *Let* $B \supseteq \min(X)$ *be a* base *for* $X$, *where* $\min(X)$ *is the set of all the minimal elements of* $X$ *w.r.t.* $\prec$. *A* rank function $r : init \to X$ *maps each initial state to a rank in* $X$. *We extend the notion of a rank to initial traces as follows. Given an initial trace* $\tau = \tau(\sigma_0)$, *we define its rank to be the rank of* $\sigma_0$. *We refer to states* $\sigma_0$ *such that* $r(\sigma_0) \in B$ *as the* base states. *Similarly, (initial) traces whose ranks are in* $B$ *are called* base traces.

In our analysis, ranks range over $X = \mathbb{N}^m$ (for some $m \in \mathbb{N}^+$), with $\prec$ defined by the lexicographic order. Ranks let us abstract away data inside the initial execution states which does *not* affect the worst-case bound on the trace length. For example, the length of traces of the binary counter program (Fig. 1) is completely agnostic to the actual content of the array at the initial state. The only parameter that affects its trace length is the array size, and not which integers are stored inside it. Hence, a suitable rank function in this example maps an initial state to its array length. This is despite the fact that the execution does depend on the content of the array, and, in particular, the number of remaining iterations from an intermediate state within the execution depends on it. The partial order $\prec$ and the base set $B$ will be used to define the recurrence formula as we explain in the sequel.

We will assume from now on that $(X, \prec, B)$, as well as the rank function, are fixed, and can be understood from context. The rank function $r$ induces a complexity function $comp_x : X \to \mathbb{N} \cup \{\infty\}$ over ranks, defined as follows.

**Definition 4 (Complexity over ranks).** *The complexity function over ranks, $comp_x : X \to \mathbb{N} \cup \{\infty\}$, is defined by:*

$$comp_x(\rho) = \max\{comp_s(\sigma_0) \mid r(\sigma_0) \preceq \rho \wedge \sigma_0 \in init\}$$

The definition ensures that for every initial state $\sigma_0 \in init$, we can compute (an upper bound on) its time complexity based on its rank, as follows: $comp_s(\sigma_0) \leq comp_x(r(\sigma_0))$. The complexity of $\rho$ takes into account all states with $r(\sigma) \preceq \rho$ and not only those with rank exactly $\rho$, to ensure monotonicity of $comp_x$ in the rank (i.e., if $\rho_1 \preceq \rho_2$ then $comp_x(\rho_1) \leq comp_x(\rho_2)$). Our approach is targeted at extracting a recurrence relation for $comp_x$.

#### 3.2 Complexity decomposition by partitioned simulation

In order to express the length of a trace in terms of the lengths of traces of lower ranks, we use a *squeezer* that maps states from the original trace to states of lower-rank traces and (implicitly) induces a correspondence between the original trace and the lower-rank trace(s). For now, we do not require the squeezer to decrease the rank of the trace; this requirement will be added later. The squeezer is accompanied by a partition function to form a *partitioned simulation* that allows a single higher-rank trace to be matched to multiple lower-rank traces such that their lengths may be correlated.

**Definition 5 (Squeezer, $\curlyvee$).** *A squeezer is a function $\curlyvee : \Sigma \to \Sigma$.*

**Definition 6.** *A function $p_d : \Sigma \to \{1, \ldots, d\}$, where $d \in \mathbb{N}^+$ is called a $d$-*partition function *if for* every *trace $\tau = \sigma_0, \sigma_1, \ldots$ it holds that $p_d(\sigma_{i+1}) \geq p_d(\sigma_i)$ for every $0 \leq i < |\tau|$.*

The partition function partitions a trace into a bounded number of *segments*, where each segment consists of states with the same value of $p_d$. We refer to the first state of a segment as a *switch state*, and to the last state of a finite segment as a *last state* (note that if $\tau$ is infinite, its last segment has no last state). In particular, this means that the initial state of a trace is a switch state. (Note that a state may be a switch state in one trace but not in another, while a last state is a last state in any trace, as long as the same partition function is considered.)

Our complexity analysis requires the squeezer to form a partitioned simulation with respect to $p_d$. Roughly, this means that the squeezer maps each segment of a trace to a (lower-rank) trace that "simulates" it. To this end, we require *all* the states $\sigma$ within a segment of a trace to be $(h, \ell)$-"stuttering", for some $h \geq \ell \geq 1$. Stuttering lets $h$ consecutive transitions of $\sigma$ be matched to $\ell$ consecutive transitions of its squeezed counterpart. If $h = \ell$, the state $\sigma$ contributes to the complexity the same number of steps as the squeezed state. Otherwise, $\sigma$ contributes $h - \ell$ additional steps, resulting in a longer trace. Recall that terminal states also have outgoing transitions (to themselves), however these transitions do not capture actual steps; they do not contribute to the complexity. Hence, stuttering also requires that "real" transitions of $\sigma$ are matched to "real" transitions of its squeezed counterpart, namely, if the latter encounter a terminal state, so must the former. For the last states of segments the requirement is slightly different as the simulation ends at the last state, and a new simulation begins in the next segment. In order to account for the transition from the last state of one segment to the first (switch) state of the next segment, last states are considered $(2, 1)$-stuttering if they are squeezed into terminal states, unless they are terminal themselves[4]. In any other case, they are considered $(1, 1)$-stuttering. The formal definitions follow.

**Definition 7 (Stuttering States).** *A non-last state $\sigma \in \Sigma$ is called a $(h, \ell)$-*stuttering *state, for $h \geq \ell \geq 1$, if: (i) $tr^{\ell}(\curlyvee(\sigma)) = \curlyvee(tr^{h}(\sigma))$; (ii) for every $i < \ell$, $tr^{i}(\curlyvee(\sigma)) \notin F$; (iii) $tr^{\ell}(\curlyvee(\sigma)) \in F$ implies that $\curlyvee(tr^{h}(\sigma)) \in F$. A last state $\sigma \in \Sigma$ is $(1,1)$-*stuttering *if $\sigma \in F$ or $\curlyvee(\sigma) \notin F$. Otherwise, it is $(2,1)$-*stuttering.

To obtain a partitioned simulation, switch states (along any trace), which start new segments, are further required to be squeezed into initial states (since our complexity analysis only applies to initial states). We denote by $\mathbb{S}_{p_d}(\tau)$ the switch states of trace $\tau$ according to partition $p_d$ and by $\mathbb{S}_{p_d}$ the switch states of *all* traces according to the partition $p_d$. Namely, $\mathbb{S}_{p_d} = init \cup \{tr(\sigma) \mid \sigma \in reach \wedge p_d(\sigma) < p_d(tr(\sigma))\}$.

**Definition 8 (Partitioned Simulation).** *We say that a squeezer $\curlyvee : \Sigma \to \Sigma$ forms a $\{(h_i, \ell_i)\}_{i=1}^{n}$-*partitioned simulation *according to $p_d$, denoted $\curlyvee \sim \mathbb{PS}_{p_d}(\{(h_i, \ell_i)\}_{i=1}^{n})$ if for every reachable state $\sigma$ we have that:*

- *$\sigma$ is $(h_i, \ell_i)$-stuttering for some $1 \leq i \leq n$, and*
- *$\sigma \in \mathbb{S}_{p_d} \Rightarrow \curlyvee(\sigma) \in init$.*

Note that Definition 7 implies that a non-terminal state may only be squeezed into a terminal state if it is the last state in its segments. When $\{(h_i, \ell_i)\}_{i=1}^{n}$ is irrelevant or clear from the context, we omit it from the notation and simply write $\curlyvee \sim \mathbb{PS}_{p_d}$.

[4] Considering a non-terminal last state that is squeezed into a terminal state as $(1, 0)$-stuttering may have been more intuitive than $(2, 1)$-stuttering, but both properly capture the discrepancy between the number of transitions in the higher and lower rank traces, and $(2, 1)$ better fits the rest of the technical development, which assumes that $h_i, \ell_i \geq 1$.

A trace squeezed by $\curlyvee \sim \mathbb{PS}_{p_d}\big(\{(h_i,\ell_i)\}_{i=1}^n\big)$ may have an unbounded number of $(h_i,\ell_i)$-stuttering states, which hinders the ability to define a recurrence relation based on the simulation. To overcome this, our complexity decomposition may use $\widehat{k} \geq 1$ to capture a common multiplicative factor of *all* the stuttering pairs, with the target of leaving only a *bounded* number of states whose stuttering exceeds $\widehat{k}$ and needs to be added separately. This will become important in Theorem 1.

**Observation 1 (Complexity decomposition)** *Let $\curlyvee \sim \mathbb{PS}_{p_d}\big(\{(h_i,\ell_i)\}_{i=1}^n\big)$, and $\widehat{k} \geq 1$. Let $\mathbb{E}_{\widehat{k}} \subseteq \{1,\ldots,n\}$ be the set of indices such that $\frac{h_i}{\ell_i} > \widehat{k}$. Then for every $\sigma_0 \in init$ we have that*

$$comp_s(\sigma_0) \leq \sum_{\sigma\in\mathbb{S}_{p_d}(\tau(\sigma_0))} \widehat{k}\cdot comp_s(\curlyvee(\sigma)) + \sum_{i\in\mathbb{E}_{\widehat{k}}}\ \sum_{\sigma\in\mathbb{K}_i(\tau(\sigma_0))} h_i - \ell_i{\cdot}\widehat{k}$$

*where $\mathbb{K}_i\big(\tau(\sigma_0)\big)$ is the multiset of $(h_i,\ell_i)$-stuttering states in $\tau(\sigma_0)$.*

In the observation, the first addend summarizes the complexity contributed by all the lower-rank traces, while using $\widehat{k}$ as an upper bound on the "inflation" of the traces. However, the states that are $(h_i,\ell_i)$-stuttering with $\frac{h_i}{\ell_i}$ that exceeds $\widehat{k}$ contribute additional $h_i - (\ell_i \cdot \widehat{k})$ steps to the complexity, and as a result, need to be taken into account separately. This is handled by the second addend, which adds the steps that were not accounted for by the first addend. While we use the same inflation factor $\widehat{k}$ across the entire trace, a simple extension of the decomposition property may consider a different factor $\widehat{k}$ in each segment. Note that the first addend always sums over a finite number of elements since the number of switch states is at most $d$ – the number of segments. If $\tau(\sigma_0)$ is finite, the second addend also sums over a finite number of elements.

Observation 1 considers the complexity function over states, and is oblivious to the rank. In particular, it does not rely on the squeezer decreasing the rank of states. Next, we use this observation as the basis for extracting a recurrence relation for the complexity function over ranks, in which case, decreasing the rank becomes important.

#### 3.3 Extraction of recurrence relations over ranks

Based on the complexity decomposition, we define recurrence relations that capture $comp_x$ — the time complexity of the initial states as a function of their ranks. To go from the complexity as a function of the actual states (as in Observation 1) to the complexity as a function of their ranks, we need to express the rank of $\curlyvee(\sigma_s)$ for a switch state $\sigma_s$ as a function of the rank of $\sigma_0$. To this end, we define $\hat{\curlyvee}$:

**Definition 9.** *Given $r$, $\curlyvee$ and $p_d$ such that $\curlyvee \sim \mathbb{PS}_{p_d}$, a function $\hat{\curlyvee} : X \times \{1,\ldots,d\} \to X$ is a* rank bounding function *if for every $\rho \in X - B$ and $1 \leq i \leq d$, if $\tau(\sigma_0)$ is an initial trace such that $r(\sigma_0) = \rho$, and $\sigma_s \in \mathbb{S}_{p_d}(\tau(\sigma_0))$ is a switch state such that $p_d(\sigma_s) = i$, the following holds:*

*(i) upper bound: $r\big(\curlyvee(\sigma_s)\big) \preceq \hat{\curlyvee}(\rho,i)$ and (ii) rank decrease: $\hat{\curlyvee}(\rho,i) \prec \rho$*

In other words, Definition 9 requires that for every non-base initial state $\sigma_0 \in \mathit{init}$ and switch state $\sigma_s$ at segment $i$ of $\tau(\sigma_0)$, we have that $r(\Upsilon(\sigma_s)) \preceq \hat{\Upsilon}(r(\sigma_0), i) \prec r(\sigma_0)$. Recall that $r(\Upsilon(\sigma_s))$ is well defined since $\Upsilon(\sigma_s)$ is required to be an initial state. The definition states that $\hat{\Upsilon}(\rho, i)$ provides an upper bound on the rank of squeezed switch states in a non-base trace of rank $\rho$. $\mathit{comp}_x(r(\Upsilon(\sigma))) \leq \mathit{comp}_x(\hat{\Upsilon}(\rho, i))$ is ensured by the monotonicity of $\mathit{comp}_x$. This definition also requires the rank of non-base traces to strictly decrease when they are squeezed, as captured by the "rank decrease" inequality.

Obtaining a rank bounding function, or even verifying that a given $\hat{\Upsilon}$ satisfies this requirement, is a challenging task. We return to this question later in this section.

These conditions allow to substitute the states for ranks in the first addend of Observation 1, and hence obtain recurrence relations for $\mathit{comp}_x$ over the (decreasing) ranks. To handle the second addend, we also need to bound the number of states whose stuttering, $\frac{h_i}{\ell_i}$, exceeds $\widehat{k}$. This is summarized by the following theorem:

**Theorem 1.** *Let $r : \mathit{init} \to X$ be a rank function, $\Upsilon : \Sigma \to \Sigma$ a squeezer and $p_d : \Sigma \to \{1, \ldots, d\}$ a partition function such that $\Upsilon \sim \mathbb{PS}_{p_d}\big(\{(h_i, \ell_i)\}_{i=1}^{n}\big)$. Let $\hat{\Upsilon} : X \times \{1, \ldots, d\} \to X$ be a rank bounding function w.r.t. $r$, $\Upsilon$ and $p_d$. If, for some $\widehat{k} \geq 1$, the number of $(h_i, \ell_i)$-stuttering states that appear along* any *non-base initial trace is bounded by a constant $b_i \in \mathbb{N}$ whenever $i \in \mathbb{E}_{\widehat{k}}$, then*

$$\mathit{comp}_x(\rho) \leq \sum_{i=1}^{d} \widehat{k} \cdot \mathit{comp}_x\big(\hat{\Upsilon}(\rho, i)\big) + \sum_{i \in \mathbb{E}_{\widehat{k}}} b_i \cdot \big(h_i - \ell_i \cdot \widehat{k}\big). \tag{6}$$

Note that a state may be $(h_i, \ell_i)$-stuttering for several $i$'s, in which case, it is sound to count it towards any of the $b_i$'s; in particular, we choose the one that minimizes $h_i - \ell_i \cdot \widehat{k}$.

**Corollary 1.** *Under the premises of Theorem 1, if $f : X \to \mathbb{N} \cup \{\infty\}$ satisfies $f(\rho) = \sum_{i=1}^{d} \widehat{k} \cdot f(\hat{\Upsilon}(\rho, i)) + \sum_{i \in \mathbb{E}_{\widehat{k}}} b_i \cdot (h_i - \ell_i \cdot \widehat{k})$ for every $\rho \in X - B$, and $\mathit{comp}_x(\rho) \leq f(\rho)$ for every $\rho \in B$, then $\mathit{comp}_x(\rho) \leq f(\rho)$ for every $\rho \in X$. We conclude that $\mathit{comp}_s(\sigma_0) \leq f(r(\sigma_0))$ for every $\sigma_0 \in \mathit{init}$.*

*Base-case complexity* In order to apply Cor. 1, we need to accompany Eq. (6) with a bound on $\mathit{comp}_x(\rho)$ for the base ranks, $\rho \in B$. Fortunately, this is usually a significantly easier task. In particular, the running time of the base cases is often constant, because intuitively, the following are correlated: (a) the rank, (b) the size of the underlying data structure, and (c) the number of iterations. In this case, symbolic execution may be used to obtain bounds for base cases (as we do in our work). In essence, any method that can yield a closed-form expression for the complexity of the base cases is viable. In particular, we can apply our technique on the base case as a subproblem.

#### 3.4 Establishing the requirements of the recurrence relations extraction

Theorem 1 defines a recurrence relation from which an upper bound on the complexity function, $\mathit{comp}_x$, can be computed (Cor. 1). However, to ensure correctness, the premises of Theorem 1 must be verified. The requirement that $\curlyvee \sim \mathbb{PS}_{p_d}(\{(h_i, \ell_i)\}_{i=1}^n)$ (see Definition 8) may be verified *locally* by examining individual (reachable) states: for any (reachable) state $\sigma$, the check for $(h_i, \ell_i)$-stuttering and switch states can, and should, be done in tandem, and require only observing at most $\max_i h_i$ transition steps from $\sigma$ and $\max_i \ell_i$ from $\curlyvee(\sigma)$. In contrast, the property required of $\hat{\curlyvee}$ is *global*: it requires $\hat{\curlyvee}(\rho, i)$ to provide an upper bound on the rank of *any* squeezed switch state that may occur in *any* position along *any* non-base initial trace whose initial state has rank $\rho$. Similarly, the property required of the bounds $b_i$ is also *global*: that the number of $(h_i, \ell_i)$-stuttering states along *any* non-base initial trace is at most $b_i$. It is therefore not clear how these requirements may be verified in general. We overcome this difficulty by imposing additional restrictions, as we discuss next.

**Establishing bounds on the number of occurrences of stuttering states** Bounds on the number of occurrences *per trace* that are sound *for every trace* are difficult to obtain in general. While clever analysis methods exist that can do this kind of accounting, we found that a stronger, simpler condition applies in many cases:

- For every $\sigma \in \mathit{reach}$, either:
  - $\sigma$ is $(h_i, \ell_i)$-stuttering with $\frac{h_i}{\ell_i} \le \widehat{k}$; <u>or</u>
  - $\sigma$ is $(h_i, \ell_i)$-stuttering (with $\frac{h_i}{\ell_i} > \widehat{k}$), *and* either $\sigma$ is a <u>switch state</u> or $tr^{h_i}(\sigma)$ is a <u>last state</u>.

This restricts these cases to occur only at the beginnings and ends of segments. It implies a total bound of $2d\cdot \max_i(h_i - \ell_i\cdot\widehat{k})$ on the "surplus" of any trace, therefore, we substitute this expression for the rightmost sum in Eq. (6).

**Validating a rank bounding function** The definition of a rank bounding function (Definition 9) encapsulates two parts. Part (ii) ensures that the rank decreases: $\hat{\curlyvee}(\rho, i) \prec \rho$ for every $\rho \in X - B$. Verifying that this requirement holds does not involve any reasoning about the states, nor traces, of the transition system. Part (i) ensures that $\hat{\curlyvee}$ provides an upper bound on the rank of squeezed switch states. Formally, it requires that $r(\curlyvee(\sigma_s)) \preceq \hat{\curlyvee}(r(\sigma_0), i)$ for every switch state $\sigma_s$ in segment $i \in \{1, \dots, d\}$ along a trace that starts from a non-base initial state $\sigma_0$. Namely, it relates the rank of the squeezed switch state, $\curlyvee(\sigma_s)$, to the rank of the initial state, $\sigma_0$, where no bound on the length of the trace between the initial state $\sigma_0$ and the switch state $\sigma_s$ is known a priori. As such, it involves global reasoning about traces. We identify two cases in which such reasoning may be avoided: (i) The partition $p_d$ consists of a single segment (i.e., $d = 1$); or (ii) The rank function extends to *any* state (and not just the initial states), while being preserved by $tr$. In both of these cases, we are able to verify the correctness of $\hat{\curlyvee}$ locally.

*A single segment.* In this case, the only switch state along a trace is the initial state, and hence the upper-bound requirement of $\hat{\curlyvee}$ boils down to the requirement that for every $\sigma_0 \in \mathit{init}$ such that $r(\sigma_0) \in X - B$, we have that $r(\curlyvee(\sigma_0)) \preceq \hat{\curlyvee}(r(\sigma_0), 1)$.

**Lemma 1.** *Let $r$, $\curlyvee$ and $p_1 : \Sigma \to \{1\}$ such that $\curlyvee \sim \mathbb{PS}_{p_1}$. Then $\hat{\curlyvee} : X \times \{1\} \to X$ satisfies the upper-bound requirement of a rank bounding function if and only if $r(\curlyvee(\sigma_0)) \preceq \hat{\curlyvee}(r(\sigma_0), 1)$ for every $\sigma_0 \in \mathit{init}$ such that $r(\sigma_0) \in X - B$.*

*Rank preservation.* Another case in which the upper-bound property of $\hat{\Upsilon}$ may be verified locally is when the $r$ can be extended to *all* states while being preserved by $tr$:

**Definition 10.** *A function* $\hat{r} : \Sigma \to X$ extends *the rank function* $r : init \to \Sigma$ *if* $\hat{r}$ *agrees with* $r$ *on the initial states, i.e.,* $\hat{r}(\sigma_0) = r(\sigma_0)$ *for every initial state* $\sigma_0 \in init$. *The extended rank function* $\hat{r}$ *is* preserved by $tr$, *if for every reachable state* $\sigma$, *we have that* $\hat{r}(tr(\sigma)) = \hat{r}(\sigma)$.

Preservation of $\hat{r}$ by $tr$ ensures that all states along a (reachable) trace share the same rank. In particular, for a reachable switch state $\sigma_s$ that lies along $\tau(\sigma_0)$, rank preservation ensures that $\hat{r}(\sigma_s) = \hat{r}(\sigma_0) = r(\sigma_0)$ (the last equality is due to the extension property), allowing us to recover the rank of $\sigma_0$ from the rank of $\sigma_s$. Therefore, the upper-bound requirement of $\hat{\Upsilon}$ simplifies into the *local* requirement that for every reachable switch state $\sigma_s$ such that $\hat{r}(\sigma_s) \in X - B$, we have that $\hat{r}(\Upsilon(\sigma_s)) \preceq \hat{\Upsilon}(\hat{r}(\sigma_s), i)$, for every $i \in \{1, \ldots, d\}$.

**Lemma 2.** *Let* $r$, $\Upsilon$ *and* $p_d : \Sigma \to \{1, \ldots, d\}$ *such that* $\Upsilon \sim \mathbb{PS}_{p_d}$. *Suppose that* $\hat{r} : \Sigma \to X$ *extends* $r$ *and is preserved by* $tr$. *Then* $\hat{\Upsilon} : X \times \{1, \ldots, d\} \to X$ *satisfies the upper-bound requirement of a rank bounding function if and only if* $\hat{r}(\Upsilon(\sigma_s)) \preceq \hat{\Upsilon}(\hat{r}(\sigma_s), i)$ *for every reachable switch state* $\sigma_s$ *such that* $\hat{r}(\sigma_s) \in X - B$ *and for every* $i \in \{1, \ldots, d\}$.

*Remark 1.* The notion of a partitioned simulation requires a switch state $\sigma_s$ to be squeezed into an initial state. This requirement may be relaxed into the requirement that $\sigma_s$ is squeezed into a *reachable* state $\Upsilon(\sigma_s)$, provided that we are able to still ensure that the rank of (some) *initial* state $\sigma'_0$ leading to $\Upsilon(\sigma_s)$ is smaller than the rank of the trace on which $\sigma_s$ lies, and that the rank of $\sigma'_0$ is properly captured by $\hat{\Upsilon}$. One case in which this is possible, is when $r$ is extended to $\hat{r}$ that is preserved by $tr$, as in this case $\hat{r}(\Upsilon(\sigma_s)) = \hat{r}(\sigma'_0) = r(\sigma'_0)$.

This subsection described *local* properties that ensure that a given program satisfies the requirements of Theorem 1. The locality of the properties facilitates the use of SMT solvers to perform these checks automatically. This is a key step for effective application of the method.

#### 3.5 Trace-length vs. state-size recurrences with squeezers

A plethora of work exists for analyzing the complexity of programs (see Section 6 for a discussion of related works). Most existing techniques for automatic complexity analysis aim to find a recurrence relation on the length of the execution trace, relating the length of a trace from some state to the length of the remaining trace starting at its successor. These are recurrences on *time*, if you will, whereas our approach generates recurrences on the state *size* (captured by the rank). Is our approach completely orthogonal to preceding methods? Not quite. It turns out that from a conceptual point of view, our approach can formulate a recurrence on time as well, as we demonstrate in this section.

*Obtaining trace-length recurrences based on state squeezers* The key idea is to use $tr$ itself as a squeezer that squeezes each state into its immediate successor. Putting aside the initial-anchor requirement momentarily, such a squeezer forms a partitioned simulation with a single segment (i.e., $p_d \equiv 1$), in which all the states along a trace are $(1, 1)$-stuttering, except for the last one (if the trace is finite), which is $(2, 1)$-stuttering. Recall that squeezers must also preserve initial states (see Definition 8), a property that may be violated when $\curlyvee = tr$, as the successor of an initial state is not necessarily an initial state. We restore the initial-anchor property by setting $\widehat{init} = \Sigma$, i.e., every state is considered an initial state[5].

A consequence of this definition is that $comp_x$ will now provide an upper bound on the time complexity of *every* state and not only of the initial states, in terms of a rank that needs to be defined. If we further define a rank-bounding function $\hat{\curlyvee}$ we may extract a recurrence relation of the form

$$comp_x(\rho) = comp_x(\hat{\curlyvee}(\rho)) + 1$$

(we use $\hat{\curlyvee}(\rho)$ as an abbreviation of $\hat{\curlyvee}(\rho, 1)$, since this is a special case where $d = 1$).

*Defining the rank and the rank bounding function* Recall that the rank $r : \Sigma \to X$ captures the features of the (initial) states that determine the complexity. To allow maximal precision, especially since *all* states are now initial, we set $X$ to be the set of *states* $\Sigma$, and define $r$ to be the identity function, $r(\sigma) = \sigma$. With this definition, $comp_x$ and $comp_s$ become one. Next, we need to define $\prec$ and $B$, while ensuring that $\curlyvee$ squeezes the (non-base) initial states, which are now *all* the states, into states of a lower rank according to $\prec$. Since squeezers act like transitions now, having that $\curlyvee = tr$, they have the effect of decreasing the number of transitions remaining to reach a terminal state (provided that the trace is finite). We use this observation to define $\prec \subseteq \Sigma \times \Sigma$. Care is needed to ensure that $(\Sigma, \prec)$ is well-founded, i.e., every descending chain is finite, even though the program may *not* terminate. Here is the definition that achieves this goal:

$$\sigma_1 \prec \sigma_2 \;\Leftrightarrow\; comp_s(\sigma_1) < comp_s(\sigma_2) \tag{7}$$

Since $\curlyvee = tr$ does not decrease $comp_s$ for states that belong to infinite (non-terminating) traces ($comp_s(\curlyvee(\sigma)) = comp_s(\sigma) = \infty$, hence $\curlyvee(\sigma) \not\prec \sigma$), they must be included in $B$, together with the terminal states, which are minimal w.r.t. $\prec$. Namely, $B = F \cup \{\sigma \mid comp_s(\sigma) = \infty\}$. Technically, this means that the base of the recurrence needs to define $comp_x$ for these states.

The final piece in the puzzle is setting $\hat{\curlyvee} = tr$. Since $\curlyvee \sim \mathbb{PS}_{p_d}\big(\{(1, 1), (2, 1)\}\big)$ (when $\widehat{init} = \Sigma$), where the number of $(2, 1)$-stuttering states that appear along any non-base initial trace is bounded by 1, we may use Theorem 1, setting $\widehat{k} = 1$, to derive the following recurrence relation, which reflects induction over time:

$$comp_x(\sigma) = comp_x(tr(\sigma)) + 1.$$

[5] In fact, it suffices to consider $\widehat{init} = reach$, in which case we may be able to take advantage of information from static analyses

The formulation above represents a degenerate, naïve, choice of ingredients for the sake of a theoretical construction, whose purpose is to lay the foundation for a general framework that takes its strengths from both induction over time and induction over rank. This construction does not exploit the full flexibility of our framework. In particular, ranking functions obtained from termination proofs, as used in [5], may be used to augment the rank in this setting. Further, invariants inferred from static analysis can be used to refine the recurrences.

### 4 Synthesis

So far we have assumed that the rank function $r$, partition function $p_d$, squeezer $\curlyvee$ and a rank bounding function $\hat{\curlyvee}$ are all readily available. Clearly, they are specific to a given program. It would be too tedious for a programmer to provide these functions for the analysis of the underlying complexity. In this section we show how to automate the process of obtaining $(r, p_d, \curlyvee, \hat{\curlyvee})$ for a class of typical looping programs. We take advantage of the fact that these components are much more compact than other kinds of auxiliary functions commonly used for resource analysis, such as monotonically decreasing measures used as ranking functions. For example, a ranking function for the binary counter program shown in Fig. 1 is:

$$m(n, i, c) = \left( n \cdot \sum_{j=0}^{n-1} 2^j \cdot c[j] \right) + (2^i - 1) + (n - i)$$

whereas the rank, partition, $\curlyvee$ and $\hat{\curlyvee}$ are

$$\begin{array}{ll} r(n, i, c) = n & \curlyvee(n, i, c) = \big(n - 1, (i \geq n) \mathbin{?} i - 1 : i, c[: n - 1]\big) \\ \hat{\curlyvee}(\rho) = \rho - 1 & p_d(n, i, c) = (i \geq n \mid\mid c[n - 1]) \mathbin{?} 2 : 1 \end{array}$$

This enables the use of a relatively naïve enumerative approach of multi-phase generate-and-test, employing some early pruning to discard obviously non-qualifying candidates.

#### 4.1 SyGuS

The generation step of the synthesis loop applies syntax guided synthesis (SyGuS [7]). Like any other SyGuS method, defining the underlying grammars is more art than science. It should be expressive enough to capture the desired terms, but strict enough to effectively bound the search space.

*Ranks* are taken from $\mathbb{N}^m$ where $m \in \{1, 2, 3\}$ and $\prec$ is the usual lexicographic order. The rank function $r$ comprises of one expression for each coordinate, constructed by adding / subtracting integer variables and array sizes. Boolean variables are not used in rank expressions.

*Partition functions* $p_d$. Our implementation currently supports a maximum number of two segments. This means that the partition function only assigns the values 1 and 2, and we synthesize it by generating a condition over the program's variables, *cond*, that selects between them: $p_d(\sigma) = cond(\sigma) \mathbin{?} 2 : 1$. Handling up to two segments is *not* an inherent limitation, but we found that for typically occurring programs, two segments are sufficient.

*Squeezers* $\curlyvee$ are the only ingredient that requires substantial synthesis effort. We represent squeezers as small loop-free imperative programs, which are natural for representing state transformations. We use a rather standard syntax with 'if-then-else' and assignments, plus a `remove-adjust` operation that removes array entries and adjusts indices relating to them accordingly. .

*Rank bounding functions* $\hat{\curlyvee}$. With a well-chosen squeezer $\curlyvee$, it suffices to consider quite simple rank bounds for the mini-traces. Hence, the rank-bounds defined by $\hat{\curlyvee}$ are obtained by adding, subtracting and multiplying variables with small constants (for each coordinate of the rank). Similar to the choice of ranks, targeting simple expressions for $\hat{\curlyvee}$ helps reduce the complexity of the final recurrence that is generated from the process.

#### 4.2 Verification

For the sake of verifying the synthesized ingredients, we fix a set $\{h_i, \ell_i\}$ of stuttering shapes, and check the requirements of Theorem 1 as discussed in Section 3.4. In particular, we check that $p_d$ is weakly monotone, i.e., that $cond$ cannot change from true to false in any step of $tr$. Note that some of the properties may be used to discriminate some of the ingredients independent of the others. For example, the simulation requirement only depends on $\curlyvee$ and $p_d$.

*Unbounded verification* Once candidates pass a preliminary screening phase, they are verified by encoding the program and all the components $r, p_d$, $\curlyvee$, $\hat{\curlyvee}$ as first-order logic expressions, and using an SMT solver (Z3 [13]) to verify that the requirements are fulfilled for all traces of the program.
As mentioned in Section 3.4, all the checks are local and require observing a bounded set of steps starting from a given $\sigma$. The only facet of the criteria that is difficult to encode is the fact they are required of the reachable states (and not any state). Of course, if we are able to ascertain that these are met for *all* $\sigma \in \Sigma$, including unreachable states, then the result is sound. However, for some programs and squeezers, the required properties (esp., simulation) do not hold universally, but are violated by unreachable states. To cope with this situation without having to manually provide invariants that capture properties of the reachable states, we use a CHC solver, Spacer [23], which is part of Z3, to check whether all the reachable states in the unbounded-state system induced by the input program satisfy these properties. This can be seen as a reduction from the problem of verifying the premises of Theorem 1 to that of verifying a safety property.

### 5 Empirical Evaluation

We implemented our complexity analyzer as a publicly available tool, SqzComp, that receives a program in a subset of C and produces recurrence relations. SqzComp is written in C++, using the Z3 C++ API [13], and using Spacer [23] via its SMTLIB2-compatible interface. Since our squeezers may remove elements from arrays, we initially encoded arrays as SMT sequences. However, we found that it is beneficial to

| Description | Real complexity | Inferred bound CoFloCo | Inferred bound SqzComp | SqzComp Time | SqzComp $d$ |
|---|---|---|---|---|---|
| array: max value | $O(\|A\|)$ | $O(\|A\|)$ | $O(\|A\|)$ | $< 1$ sec | 1 |
| array: min value | $O(\|A\|)$ | $O(\|A\|)$ | $O(\|A\|)$ | $< 1$ sec | 1 |
| array: find first | $O(\|A\|)$ | $O(\|A\|)$ | $O(\|A\|)$ | $< 1$ sec | 1 |
| array: find last | $O(\|A\|)$ | $O(\|A\|)$ | $O(\|A\|)$ | $< 1$ sec | 1 |
| array: is-sorted | $O(\|A\|)$ | $O(\|A\|)$ | $O(\|A\|)$ | $< 1$ sec | 1 |
| array: longest asc. prefix | $O(\|A\|)$ | $O(\|A\|)$ | $O(\|A\|)$ | $< 1$ sec | 1 |
| array: binary search | $O(\log(\|A\|))$ | $O(\log(\|A\|))$ | $O(\log(\|A\|))$ | $< 1$ sec | 1 |
| gcd | $\max(x, y)$ | $O(x + y)$ | $O(x + y)$ | $< 1$ sec | 1 |
| two-phase loop 1 | $O(2n - 2x + y)$ | $O(2n - 2x + y)$ | $O(2n + 2y)$ | $< 1$ sec | 1 |
| two-phase loop 2 | $O(n - x + m - y)$ | $O(n - x + m - y)$ | $O(n - x + m - y)$ | $< 1$ sec | 1 |
| two-phase loop 3 | $O(n)$ | $O(n)$ | $O(n)$ | $< 1$ sec | 1 |
| two-phase loop 4 | $O(2n - x - z)$ | $O(2n - x - z)$ | $O(2n)$ | $< 1$ sec | 1 |
| multi-path loop 1 | $O(n)$ | $O(3n)$ | $O(n)$ | $< 1$ sec | 1 |
| multi-path loop 2 | $O(n)$ | $O(n)$ | $O(n)$ | $< 1$ sec | 1 |
| multi-path loop 3 | $O(n)$ | $O(n)$ | $O(n)$ | $< 1$ sec | 1 |
| tricky init loop | $O(z)$ | $O(z)$ | $O(z)$ | 4 min | 1 |
| nested loop 1 | $O(\|x - y\|)$ | $O(\|x - y\|)$ | $O(x + y)$ | $< 1$ sec | 1 |
| nested loop 2 | $O(a^2)$ | $O(a^2)$ | $O(a^2)$ | 16 min | 1 |
| context sensitive loop | $O(\max(n - m, m))$ | $O(\max(n - m, m))$ | $O(n)$ | 7 min | 1 |
| binary counter | $O(2^{n+1})$ | $\infty$ | $O(2^{n+1})$ | 34 min | 2 |
| subsets | $O(\binom{n-m}{k})$ | $\infty$ | $O(\binom{n-m}{k})$ | 50 min | 2 |
| monotone sequences | $O(\binom{n}{k})$ | $\infty$ | $O(\binom{n}{k})$ | 50 min | 2 |

**Table 1.** Experimental results. In array programs, $A$ denotes an array. $x, y, z, n, m, k, a$ are integer variables.

restrict squeezers to only remove the first or last elements of an array, resulting in a more efficient encoding with the theory of arrays. For the base case of generated recurrences, we use the symbolic execution engine KLEE [11] to bound the total number of iterations by a constant.

### 5.1 Experiments

We evaluated our tool, SqzComp, on a variety of benchmark programs taken from [16], as well as three additional programs: the binary counter example from Section 2, a subsets example, described in Section 5.2, and an example computing monotone sequences. These examples exhibit intricate time complexities. From the benchmark suite of [16] we filtered out non-deterministic programs, as well as programs that failed syntactic constraints that our frontend cannot currently handle. We compared SqzComp to CoFloCo [16]—the state of the art tool for complexity analysis of imperative programs.

Table 1 summarizes the results of our experiments. The first column presents the name of the program, which describes its characteristics (each of the "two-phase loop" programs consists of a loop with an if statement, where the branch executed changes starting from some iteration). The second column specifies the real complexity, while the following two columns present the bounds inferred by SqzComp and by CoFloCo, respectively. (For SqzComp, the reported bounds are the solutions of the recurrences

```
1 void subsets(uint n, uint k, uint m) {
2   uint I[k]; int j = 0; bool f = true;
3   while (j >= 0) {
4     if (j >= k)    /*start left scan*/{f=false; j--;}
5     else if (j==0 && f)       /*init*/{f=true;I[0]=m;j++;}
6     else if (f)         /*right fill*/{f=true;I[j]=I[j-1]+1;j++;}
7     else if (I[j]>=n-k+j)/*left scan*/{f=false; j--;}
8     else          /*start right fill*/{f=true; I[j]=I[j]+1;j++;}
9  }}
```

```
squeezer(uint I[], uint n, uint k, uint m, int j, bool f) {
  if      (I[0]==m  &&  j>0) { m++; remove I[0]; k--; j--; }
  else if (I[0]==m)          { m++; remove I[0]; k--;      }
  else                       { m++;                        }
}
```

**Fig. 4.** An example program that produces all subsets of $\{m, \ldots, n-1\}$ of size $k$; below is the synthesized squeezer.

output by the tool.) The fourth and fifth columns present the analysis running time, respectively the number of segments used in the analysis, of SqzComp.

CoFloCo's analysis time is always in the order of magnitude of 0.1 second, whether it succeeds to find a complexity bound or not. Our analysis is considerably slower, mostly due to the naïve implementation of the synthesizer. When both CoFloCo and SqzComp succeed, the bounds inferred by CoFloCo are sometimes tighter.

However, SqzComp manages to find tight complexity bounds for the new examples, which are not solved by CoFloCo, and to the best of our knowledge, are beyond reach of existing tools. (We also encoded the new examples as OCaml programs and ran the tool of [20] on them, and it failed to infer bounds.)

#### 5.2 Case study: Subsets example

This subsection presents one challenging example from our benchmarks, the subsets example, and the details of its complexity analysis. Notably, our method is able to infer a binomial bound, which is asymptotically tight.

The code, shown in Fig. 4, iterates over all the subsets of {`m,...,n-1`} of size `k`. The "current" subset is maintained in an array `I` whose length is `k`, and which is always sorted, thus avoiding generating the same set more than once. The first $k$ iterations of the loop fill the array with values {`m,m+1,...,m+k-1`}, which represent the first subset generated. This is taken care of by the branches at lines 5, 6 that perform a "right fill" phase, filling in the array with an ascending sequence starting from `m` at `I[0]`. Once the first $k$ iterations are done, `j` reaches the end of the array (`j=k`) and so the next iteration will execute line 4, turning off the flag `f`, signifying that the array should now be scanned leftwards. In each successive iteration, `j` is decreased, looking for the rightmost element that can be incremented. For example, if $n = 8, I = [2, 6, 7]$, this rightmost element is $I[0] = 2$. After that element is incremented, the flag `f` is turned on again, completing the "left scan" phase and starting a "right fill" phase.

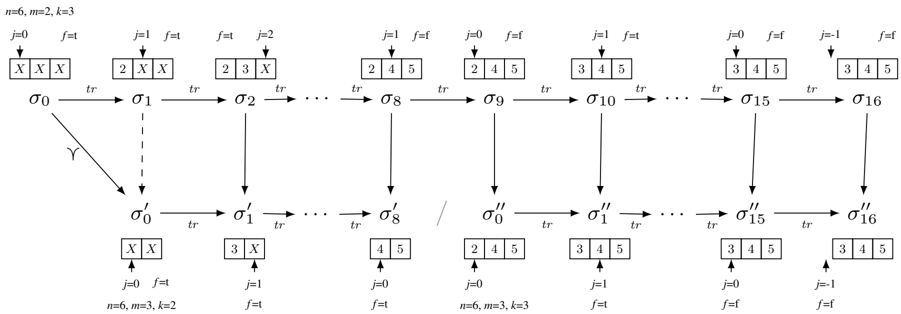

**Fig. 5.** An illustration of the 2-partitioned simulation for the subsets example. In the univariate case, the rank of the upper trace is $n - m$ and that of the lower traces is $n - m - 1$. In the multivariate case, the upper trace is of rank $(n - m, k)$, lower traces of ranks $(n - m - 1, k - 1)$, $(n - m - 1, k)$.

*A univariate recurrence* Consider the rank function $r(I, n, k, m, j, f) = n - m$, defined with respect to $(\mathbb{N}, <)$, and the squeezer shown below the program in Fig. 4. The squeezer observes the first element of the array: if it is equal to $m$ (the lower bound of the range), it removes it from the array, shrinking its size ($k$) by one. It then adjusts the index $j$ to keep pointing to the same element; unless $j = 0$, in which case that element is removed. This squeezer forms a 2-partitioned simulation, as illustrated by the traces in Fig. 5. All states are $(1, 1)$-stuttering, except for $\sigma_0$, which is $(2, 1)$-stuttering, as caused by the removal of $I[0]$ when $j = 0$. The rank bounding function is $\hat{\Upsilon}(i, \rho) = \rho - 1$ for $i \in \{1, 2\}$. We therefore obtain the following recurrence relation:

$$comp_x(\rho) \leq 1 + comp_x(\rho - 1) + comp_x(\rho - 1).$$

The base of the recurrence is $comp_x(0) = 1$, leading to the solution $comp_x(\rho) \leq 2^{\rho+1} - 1$. This means that for an initial state, $comp_s(I, n, k, m, 0, \mathsf{true}) \leq comp_x(n - m) \leq 2^{n-m+1} - 1$.

*A multivariate recurrence* Consider an alternative rank definition $r(I, n, k, m, j, f) = (n - m, k)$ defined with respect to $(\mathbb{N} \times \mathbb{N}, <)$, where '$<$' denotes the lexicographic order, together with the same squeezer and partition as before. The rank bounding function is now $\hat{\Upsilon}\big((\rho_1, \rho_2), i\big) = \begin{cases} (\rho_1 - 1, \rho_2 - 1) & i = 1 \\ (\rho_1 - 1, \rho_2) & i = 2 \end{cases}$. The corresponding recurrence relation is:

$$comp_x(\rho_1, \rho_2) \leq 1 + comp_x(\rho_1 - 1, \rho_2 - 1) + comp_x(\rho_1 - 1, \rho_2)$$

with base $comp_x(0, \_) = 1$, resulting in the solution $comp_x(\rho_1, \rho_2) \leq \binom{\rho_1+2}{\rho_2}$. That is, for an initial state, $comp_s(I, n, k, m, 0, \mathsf{true}) \leq comp_x(n - m, k) \leq \binom{n-m+2}{k}$.

Interestingly, this example demonstrates that the same squeezer may yield different recurrences, when different ranks (and rank bounding functions) are considered. It also demonstrates a case where different segments of a trace are mapped to mini-traces of a different rank.

## 6 Related Work

This section focuses on exploring existing methods for *static* complexity analysis of *imperative* programs. Dynamic profiling and analysis [26] are a separate research area, more related to testing, and generally do not provide formal guarantees. We further focus on works that determine *asymptotic* complexity bounds, and use the number of iterations executed as their cost model; we refrain from thoroughly covering previous techniques that analyze complexity at the instruction level.

*Static cost analysis* The seminal work of [28] defined a two steps meta-framework where recurrence relations are extracted from the underlying program, and then analyzed to provide closed-form upper bounds. Broadly speaking, cost relations are a generalized framework that captures the essence of most of the works mentioned in this section.

[4] and [16] infer cost relations of imperative programs written in Java and C respectively. Cost relations resemble somewhat limited C procedures: They are capable of recursive calls to other cost relations, and they can handle non-determinism that arises either as a consequence of direct `nondet()` in the program, or as a result of inherent imprecision of static analysis. They define for every basic block of the program its own cost relation function, and then form chains according to the control flow graph of the program. They use numerical abstract domains to support a context sensitive analysis of whether a chain of visits to specific basic blocks is feasible or not. Once all infeasible chains are removed, disjunctive analysis determines an overall approximation of the heaviest chain, representing the max number of iterations.

[19] uses multiple counter instrumentation that are automatically inserted in various points in the code, initialized and incremented. These ghost counters enable to infer an overall complexity bound by applying appropriate abstract interpretation handling numeric domains. [18] and [17] apply code transformations to represent multi-path loops and nested loops in a canonical way. Then, paths connecting pairs of "interesting" code points $\pi_1, \pi_2$ (loop headers etc.) are identified, in a way that satisfies some properties. For instance, $\pi_1$ is reached twice *without* reaching $\pi_2$. The path property induces progress invariants, which are then analyzed to infer the overall complexity bound.

[24] define an abstraction of the program to a *size-change-graph*, where transition edges of the control flow graph are annotated to capture sound over-approximation relations between integer variables. The graph is then searched for infinitely decreasing sequences, represented as words in an $\omega$-regular language. This representation concisely characterizes program termination. [29] then harnesses the size-change abstraction from [24] to analyze the complexity of imperative programs. First, they apply standard program transformations like pathwise analysis to summarize inner nested loops. Then, they heuristically define a set of scalar rank functions they call norms. These norms are somewhat similar to our rank function in the sense that they help to abstract away program parts that do not effect its complexity. The program is then represented as a size-change graph, and multi-path contextualization [25] prunes subsequent transitions which are infeasible.

[8] introduces *difference constraints* in the context of termination, to bound variables $x'$ in current iteration with some $y$ in previous iteration plus some constant $c$:

$x' \leq y + c$. [27] extends difference constraints to complexity analysis. Indeed, it is quite often the case that ideas from the area of program termination are assimilated in the context of complexity analysis and vice versa. They exploit the observation that typical operations on loop counters like increment, decrement and resets are essentially expressible as difference constraints. They design an abstraction based on the domain of difference constraints, and obtain relevant invariants which are then used in determining upper bounds. [10] is very similar, only that it represents a program as an integer transition system and allows nonlinear numerical constraints and ranking functions. As we mentioned earlier, all of these approaches are based on identifying the progress of executions over time, characterizing the progress between two given points in the program. In contrast, our approach allows to reason over state size and compares whole executions.

*Squeezers.* The notion of squeezers was introduced by [22] for the sake of safety verification. As discussed in Section 1, the challenges in complexity analysis are different, and require additional ingredients beyond squeezers. [15,1,2] introduce *well structured transition systems*, where a well-quasi order (wqo) on the set of states induces a simulation relation. This property ensures decidability of safety verification of such systems (via a backward reachability algorithm). Our use of squeezers that decrease the rank of a state and induce a sort of a simulation relation may resemble the wqo of a well structured transition system. However, there are several key differences: we do not require the order (which is defined on ranks) to be a wqo. Further, we do not require a simulation relation between *any* states whose ranks are ordered, only between a state and its squeezed counterpart. Notably, our work considers complexity analysis rather than safety verification.

## 7 Conclusion

This work introduces a novel framework for run-time complexity analysis. The framework supports derivation of recurrence relations based on inductive reasoning, where the form of induction depends on the choice of a squeezer (and rank bounding function). The new approach thus offers more flexibility than the classical methods where induction is coupled with the time dimension. For example, when the rank captures the "state size", the approach mimics induction over the space dimension, reasoning about whole traces, and alleviating the need to describe the intricate development of states over time. We demonstrate that such squeezers and rank bounding functions, which we manage to synthesize automatically, facilitate complexity analysis for programs that are beyond reach for existing methods. Thanks to the simplicity and compactness of these ingredients, even a rather naïve enumeration was able to find them efficiently.

**Acknowledgements.** The research leading to these results has received funding from the European Research Council under the European Union's Horizon 2020 research and innovation programme (grant agreement No [759102-SVIS]). This research was partially supported by the United States-Israel Binational Science Foundation (BSF) grant No. 2016260 and 2018675, the Israeli Science Foundation (ISF) grants No. 1996/18, 1810/18, 243/19 and 2740/19, and the Pazy Foundation.
